# What Goes in Must Come out: Testing for Biases in Molecular Analysis of Arbuscular Mycorrhizal Fungal Communities

**DOI:** 10.1371/journal.pone.0109234

**Published:** 2014-10-02

**Authors:** T. E. Anne Cotton, Alex J. Dumbrell, Thorunn Helgason

**Affiliations:** 1 Department of Biology, University of York, York, United Kingdom; 2 School of Biological Sciences, University of Essex, Colchester, United Kingdom; Institute for Sustainable Plant Protection, C.N.R., Italy

## Abstract

Arbuscular mycorrhizal (AM) fungi are widely distributed microbes that form obligate symbioses with the majority of terrestrial plants, altering nutrient transfers between soils and plants, thereby profoundly affecting plant growth and ecosystem properties. Molecular methods are commonly used in the study of AM fungal communities. However, the biases associated with PCR amplification of these organisms and their ability to be utilized quantitatively has never been fully tested. We used Terminal Restriction Fragment Length Polymorphism (TRFLP) analysis to characterise artificial community templates containing known quantities of defined AM fungal genotypes. This was compared to a parallel *in silico* analysis that predicted the results of this experiment in the absence of bias. The data suggest that when used quantitatively the TRFLP protocol tested is a powerful, repeatable method for AM fungal community analysis. However, we suggest some limitations to its use for population-level analyses. We found no evidence of PCR bias, supporting the quantitative use of other PCR-based methods for the study of AM fungi such as next generation amplicon sequencing. This finding greatly improves our confidence in methods that quantitatively examine AM fungal communities, providing a greater understanding of the ecology of these important fungi.

## Introduction

Arbuscular mycorrhizal (AM) fungi form the most abundant symbiosis on earth, colonising the roots of approximately two thirds of terrestrial plants [1], [2]. In addition to being extremely common, they are also highly influential in ecosystems, affecting plant growth and communities [3]–[6], driving nutrient cycles [7], [8], determining soil properties [8], [9] and interacting with many other soil organisms [10], [11]. However, AM fungi are extremely difficult to study by non-molecular approaches due to their cryptic morphology and complex growth requirements. Thus, molecular methods have revolutionised our understanding of these organisms and are amongst the most promising tools for their study [12], [13].

One of the most widely used DNA-based methods is the analysis of Terminal Restriction Fragment Length Polymorphisms (TRFLPs; e.g. refs [14]–[17]). TRFLP analysis involves polymerase chain reaction (PCR) amplification of environmental DNA extracts using fluorescently labelled primers, restriction digestion of the resulting terminally labelled amplicons and measurement of the sizes of the terminal restriction fragments (TRFs) produced [18]. This produces community ‘fingerprints’ for samples analysed that can be compared in terms of diversity and composition. The use of TRFLPs alone does not produce sequences of the organisms in a community, but if used in conjunction with sequence databases, putative identities may be assigned to TRFs, allowing their identification [13], [19].

Many studies have used TRFLPs and other molecular methods such as next generation sequencing (NGS) to study AM fungal communities (e.g. refs [14]–[17], [20], [21]). Most of these studies have made the major assumption that there are minimal biases in these molecular methods which could influence the quantification of AM fungal communities. Yet these methods all involve PCR, and PCR bias, whereby different genotypes are preferentially amplified due to their nucleotide composition or amplification reaction kinetics (reviewed in ref. [22]), is therefore possible. It has been argued that PCR amplification of AM fungi with the commonly used primers AM1 and NS31 is unlikely to be biased due to the conserved nature of their primer binding regions and low variability in GC content in the resulting amplicons [14], but this has never been empirically tested. If such biases do exist, the results from such studies could only be used qualitatively, preventing more informative, quantitative analyses. However, if such biases are not present, it would allow the differentiation of communities that contain the same components, but in different relative abundances and the calculation of diversity estimates based on evenness as well as richness.

In this study, we created artificial community templates containing known ratios of characterised AM fungal genotypes and analysed them using TRFLP analysis. The relative abundances of TRFs this generated and the modelled relative abundances produced assuming no bias in the PCR and TRFLP process, were analysed and compared. This experiment thereby tested the hypothesis that TRFLP analysis accurately measures the relative differences between different AM fungal communities in terms of their composition, diversity and structure.

## Materials and Methods

### Generating community template components

Artificial community templates were produced by mixing PCR products from 18S rDNA clone libraries produced for a study of AM fungi in soybean roots [23]. In summary, total community DNA was extracted from soybean roots using a PowerPlant DNA isolation Kit (Mo Bio Laboratories, Inc., CA, USA) according to the manufacturer's instructions. Partial small subunit (SSU) ribosomal DNA fragments (*ca*. 550 bp) were amplified with the universal eukaryotic primer NS31 [24] and the AM fungal specific primer AM1 [25]. PCR products were cleaned using a QIAquick PCR Purification Kit (Qiagen Ltd, West Sussex, UK) and cloned into pGEM-T Easy Vector (Promega, WI, USA) by incubating 3µl of cleaned PCR product with 5µl of ligation buffer, 1µl T4 DNA ligase and 1µl of pGEM-T Easy Vector for 3 hours at room temperature. Vectors (4µl) were transformed into *Escherichia coli* cells by incubation with 25µl competent *E. coli* cells (DH5α; Invitrogen, Renfrewshire, UK) at 4°C for 30 minutes, followed by a heatshock of 42°C for 45 seconds and rotary incubation with 475µl Super Optimal broth with Catabolite repression (SOC) at 37°C for 1 hour. The resulting bacteria were plated onto LB-agar amended with X-gal and ampicillin. These were incubated at 37°C overnight before storing at 4°C. Putative positive transformants were amplified using primers SP6 and T7 (as described in ref. [26]), which bind to the plasmid DNA on either side of the DNA insert, producing *ca*. 725 bp amplicons containing the 18S AM fungal insert flanked by DNA derived from the plasmid. These were sequenced (Technology Facility, University of York, UK) using primer SP6 and BigDyeTM terminator cycling conditions on an ABI 3130 capillary sequencer (Applied Biosystems Inc, Applied Biosystems Inc., CA, USA). Sequences were aligned using ClustalX [27] with default parameter settings and pairwise sequence similarities were calculated. Six distinct sequence types (genotypes) were chosen to generate the artificial DNA templates created, which when combined met three criteria: 1) virtual digestion *in silico* suggested they would each produce uniquely sized TRFs with each of two enzymes commonly used in TRFLP analyses: *Alu*-I and *Hinf*-I; 2) the AM1/NS31 insert had the same orientation within the plasmid; 3) each genotype showed ≤97% pairwise sequence similarity to all other genotypes used, reflecting commonly used cut-offs for defining Operational Taxonomic Units (OTUs). These sequences have been submitted to the European Molecular Biology Laboratory (EMBL) Nucleotide Sequence Database (accession numbers KJ749695-KJ749700).

To generate sufficient genetic material to produce the templates, PCR products derived from the chosen genotypes were amplified again using the same protocol as before (with primers SP6 and T7), but with only 15 cycles to reduce the likelihood of errors. The resulting PCR products were purified using a QIAquick PCR Purification Kit (Qiagen) and divided into three separate aliquots and the concentration of DNA in each was measured in triplicate using a nanodrop-1000 spectrophotometer (NanoDrop Technologies, DE, USA).

### Creating artificial community templates

**Figure 1:**
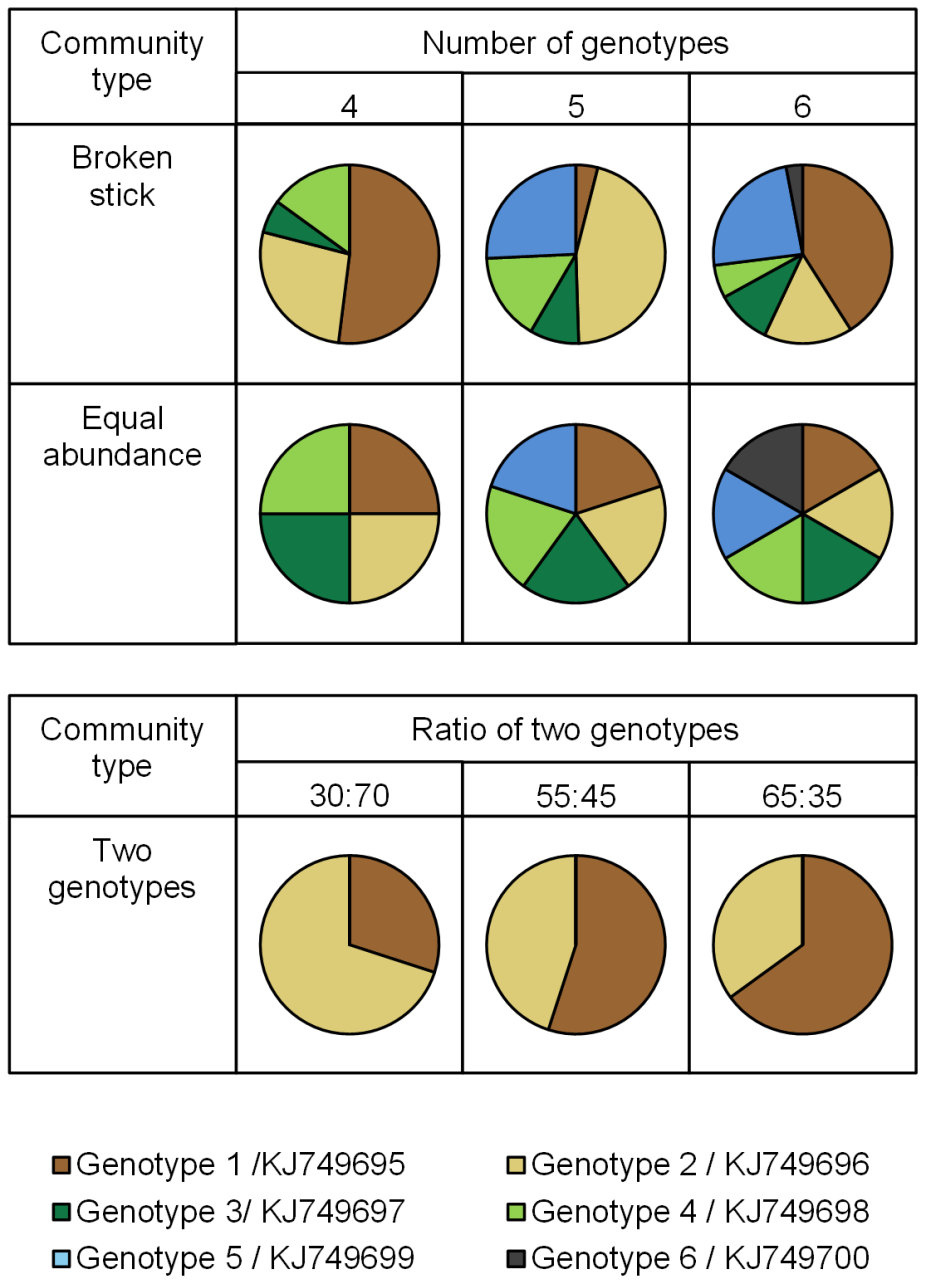
Identities and relative proportions of genotypes in each of the nine artificial community templates created. Accession numbers (KJ749695-KJ749700) refer to the European Molecular Biology Laboratory Nucleotide Sequence Database.

Six different artificial community templates were produced by combining four, five or six of the genotypes in different proportions (Figure 1). These different levels of species richness were chosen as these are typical of the number of AM fungal sequence types found in environmental samples (e.g. refs [28], [29]). ‘Equal abundance’ templates had all genotypes present in equal amounts, created to test whether particular genotypes were preferentially detected by TRFLP analysis. In ‘broken stick’ templates, the amounts of each component followed a broken stick model, designed to simulate the structure of AM fungal communities often found in environmental samples [26]. Three templates were also produced using just two genotypes in almost equal, but slightly different proportions (Figure 1) to test the resolution of TRFLP analysis. All nine templates were produced in triplicate, with each replicate produced using a different aliquot of its component genotypes.

### Experimental artificial community template analysis using TRFLPs

All artificial communities were characterised using TRFLPs. This was performed three times on each of the 27 templates to provide technical replicates to examine the repeatability of the protocol. To confirm that each genotype produced the predicted TRFs, the three aliquots of each genotype were also analysed. All templates were analysed by amplifying a 550 bp partial fragment of SSU rDNA by PCR using HotstarTaq Plus DNA polymerase (Qiagen) with primers AM1 and NS31 labelled on the 5′ end with the fluorescent markers HEX and FAM respectively (Eurofins MWG Operon, Ebersberg, Germany). The 25µl reactions contained 0.64 mM dNTPs, 0.2µM of each primer, 0.5 mg dried skimmed milk powder (to prevent PCR inhibition; Premier Interational Foods Ltd, Lincs., UK.) and the manufacturer's reaction buffer (PCR conditions: 95°C for 5 min; 40 cycles at 95°C for 30 sec, 63°C for 1 min and 72°C for 1 min; and 72°C for 10 min on a Techne TC-512 (Techne Co. Staffs., UK). PCR products were cleaned using a QIAquick PCR Purification Kit (Qiagen) and the concentrations of DNA they contained were measured using a nanodrop-1000 spectrophotometer.

Eight microlitres of each cleaned PCR product were separately digested using five units of *Hinf-*I and *Alu-*I (Promega) in a reaction volume of 15µl, with manufacturer's buffer and 2µg BSA for 15 hours. Restriction products were cleaned using a QIAquick PCR Purification Kit (Qiagen). A size standard (GS600-LIZ) was added and samples were run on an ABI 3130 genetic analyzer (Applied Biosystems Inc.). GeneMapper v4.0 software (Applied Biosystems Inc.) was used to determine peak size and quantity. Peaks greater than 100 fluorescent units in height representing TRFs longer than 100 base pairs (bp) were analysed using a bin width of 2 bp and the local southern size calling method. Peak area was used to measure TRF abundance rather than peak height, as using peak height can down-weight longer fragments as they produce wider peaks due to their longer retention time during electrophoresis [30], [31]. Before analysis, TRFs which, when present, represented an average of 5% or less of the TRFLP output were excluded to eliminate noise as in ref. [14]. As in ref. [23], only HEX labelled TRFs generated from *Alu*-I digestion and FAM labelled TRFs from *Hinf*-I digestion were further analysed, as other TRFs were predicted to be undetectable and uninformative as they were identical for all genotypes and <50 bp.

### Data analysis

#### Predicted TRFLP data

Theoretical community templates were produced by *in silico* digestion (using *Hinf-*I and *Alu-*I cutting sites) of the six distinct AM fungal genotype sequences. The resulting virtual TRFs were then combined in the same ratios as the artificial community templates. This produced nine predicted theoretical AM fungal communities with which to compare the observed artificial communities empirically analysed using TRFLPs. In addition, the size of possible under-digested terminal fragments, formed where the enzymes did not cleave the DNA at all possible cutting sites (designated ‘pseudo-TRFs’, see ref. [32]), of each fungal genotype were predicted from their sequences.

#### Compositional analysis

Principal component analysis (PCA) was used to examine whether the TRFLP protocol used could differentiate between the nine artificial communities, when analysed using ordination based on raw species abundance matrices. Covariance PCA was separately performed on the observed data from the empirically tested templates and the predicted data based on *in silico* digestion of the sequences using Minitab v.17 (Minitab Ltd., West Mids., UK). ANOVAs and Tukey's honestly significant difference tests were used to compare the templates' observed PC1 and PC2 scores to determine if they could be differentiated from each other using these community characterisation measures. Identical analyses were also performed on the predicted data.

All possible Euclidean distances between the PC scores of all possible pairs of templates from the observed data were calculated and identical calculations were also performed on the predicted data. To determine the relationship between observed and predicted TRFLP data, the two sets of distances were then compared with a Mantel test (using 10,000 randomisations). Bray Curtis dissimilarity coefficients between pairs of templates were also calculated for all predicted and observed results and similarly compared with a Mantel test, to evaluate TRFLP data as a quantitative measure of community differences.

#### Diversity analysis

Margalef's and Simpson's diversity indices were calculated from both the predicted and observed TRFs to determine how accurately the TRFLP protocol predicted the communities' α diversity levels. These indices were chosen as Margalef's index is primarily influenced by the richness of a community whereas Simpson's is primarily influenced by evenness. The relationship between predicted and observed indices were tested using linear regression.

Unless stated otherwise, all analyses were conducted using the R statistical language with the ‘vegan’ and ‘ade4’ libraries [33]–[35].

## Results

### Template constituents

There was little variation in length (527 bp–548 bp) of the AM1/NS31 amplicons generated and all sequences had low GC content (39.7%–41.7%), typical of AM fungal AM1/NS31 amplicons. Genotype 2 was similar to sequences from voucher specimens of Gigasporaceae whereas the remaining five genotypes were from the Glomeraceae. The AM1/NS31 amplicon of sequences differed from each other by an average of 9.7% (minimum dissimilarity 4.2%, maximum dissimilarity 16.1%). Genotypes were predicted to produce unique HEX labelled TRFs when digested with enzyme *Alu*-I and unique FAM labelled fragments with enzyme *Hinf*-I (Table 1).

**Table 1:** Predicted and observed TRF sizes using restriction enzymes *Alu*-I and *Hinf*-I.

Enzyme	Genotype identity	Predicted TRF size (bp)	Observed TRF size (bp)	Deviation of observed from predicted TRF size (bp)
*Alu*-I	Genotype 1 (KJ749695)	162.0	157.7	−4.3
	Genotype 6 (KJ749700)	309.0	302.0/303.8/305.1	−7.0/−5.2/−3.9
			309.6	
			310.6	
	Genotype 4 (KJ749698)	416.0	406.7/411.7	−9.3/−4.3
	Genotype 5 (KJ749699)	449.0	436.0/442.3/443.5	−13.0/−6.7/−5.5
	Genotype 3 (KJ749697)	469.0	455.6/459.5/463.7	−13.4/−9.5/−5.3
	Genotype 2 (KJ749696)	504.0	497.0/498.3/499.2	−7.0/−5.7/−4.8
*Hinf*-I	Genotype 1 (KJ749695)	141.5	137.6	−3.9
	Genotype 5 (KJ749699)	160.5	154.4	−6.1
	Genotype 4 (KJ749698)	190.5	188.2	−2.3
	Genotype 2 (KJ749696)	299.5	297.4	−2.1
			503.4	
			518.9	
			522.6	
	Genotype 6 (KJ749700)	524.5	523.6	−0.9
	Genotype 3 (KJ749697)	547.0	548.1	+1.1

Genotype identities refer to those given in Figure 1 and accession numbers in the European Molecular Biology Laboratory Nucleotide Sequence Database. Predicted terminal restriction fragment (TRF) sizes are the lengths of fragments (in base pairs; bp) generated by *in silico* digestion of sequences of each genotype. Observed TRFs are those found from the empirical analysis of artificial community templates. TRFs which were produced by empirical TRFLP analysis of individual genotypes and have therefore been combined in further analyses are given in the same row. *Alu*-I TRFs refer to HEX labelled fragments and *Hinf*-I to FAM labelled fragments.

### Detection of TRFs from single genotypes

Observed TRFLP analysis of *Hinf*-I digested amplicons derived from single genotypes showed that an average of 99.2% of the total fragments produced were detected as a single peak, representing the same size fragment as that predicted by the theoretical digests. The sizes of the remaining TRFs corresponded to predicted pseudo-TRFs, which the *in silico* analysis suggested would be produced if the sequences had not fully digested (Table 1). In contrast, instead of producing one peak at approximately the size predicted by the *in silico* analysis, for all but one sequence, digestion of individual genotypes with *Alu*-I produced 2–3 similarly sized peaks of approximately their predicted TRF length, but deviating by between 3.9–13.9 bp (Table 1). When the areas of these peaks were combined, they accounted for 99.9% of the TRFs found in each profile. In all subsequent analysis involving quantifying the relative abundance of these genotypes, the peak areas for these TRFs were therefore combined (Table 1).

### Observed TRFLP data from artificial community templates

The raw TRFLP profiles for observed artificial communities contained 17 TRFs using *Alu*-I (Table 1) whereas the predicted communities contained only six. This discrepancy was mainly caused by the production of multiple peaks for each TRF, and was reduced to eight after pooling based on the digestion of individual genotypes (Table 1). This included the six TRFs predicted from the theoretical analysis, and two TRFs of 310 and 311 bp, which correspond with the size of a predicted pseudo-TRF, formed by the under-digestion of genotype one, found in all templates (Figure 1; Table 1).

TRFLP analysis of individual genotypes using *Hinf*-I produced nine uniquely sized TRFs. Again, these included the six TRFs predicted from the *in silico* analysis and three fragments (503 bp, 519 bp, 523 bp) which corresponded to the sizes of predicted pseudo-TRFs (Table 1).

### Community differentiation

**Figure 2:**
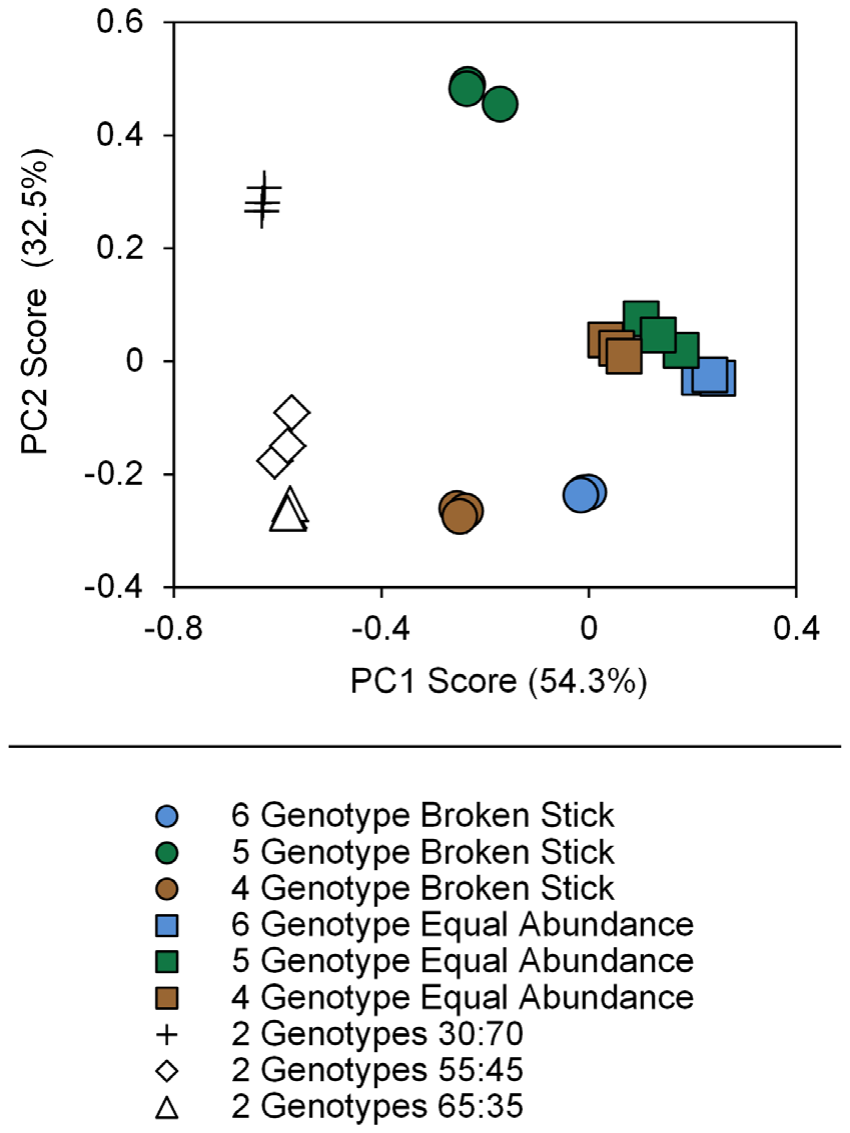
Ordination plot of principal component analysis of observed (empirically measured) TRFLP data. Mean principal component factor one (PC1) and two (PC2) scores are plotted for each template replicate. Error bars are standard errors of the means based on technical replicates (replicate PCR and digestion) but they are too small to be visible (*n* = 3).

PCA of the predicted and observed TRFLP data explained 88% and 87% of the variation in the data across the first two ordination axes (Figure 2). PC1 and PC2 scores generated from the observed TRF data were significantly different between the nine artificial community templates (Figure 2; ANOVA; PC1 scores: *F*_8,18_ = 761, *P*<0.001; PC2 scores: *F*_8,18_ =  465, *P*<0.001). Tukey's honestly significant difference tests showed all templates could be differentiated from each other using either or both their PC1 or PC2 scores (*P* values <0.05; Figure 2). Quantitative TRFLP analysis therefore allowed differentiation between all templates with the same number and identity of genotypes, but with different relative abundances. In addition, there was very little variation in PC1 and PC2 scores of the three technical replicates of each of the 27 templates, indicating a high repeatability of this method (Figure 2).

**Figure 3:**
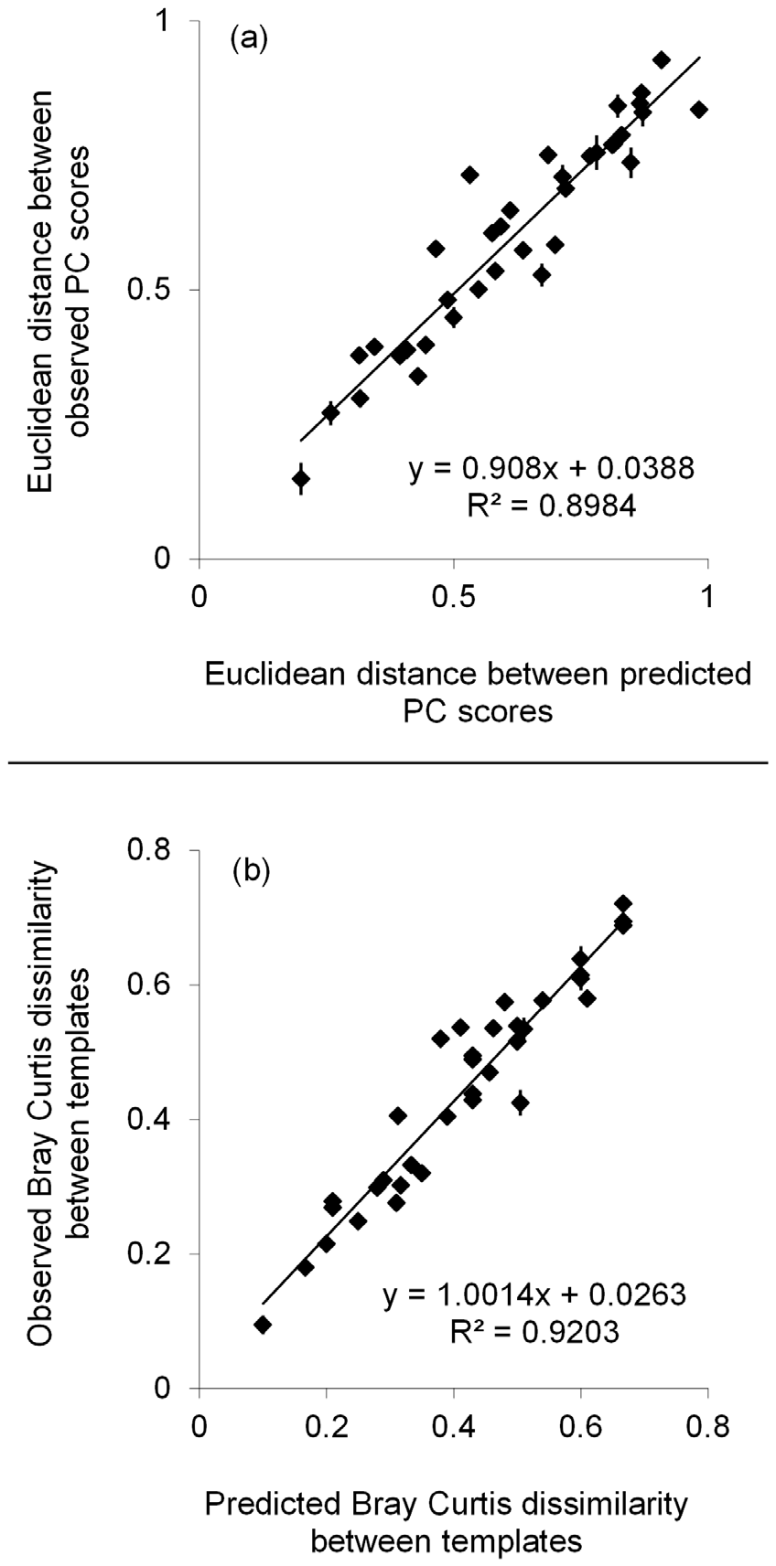
Relationship between predicted and observed differences between pairs of template communities. Euclidean distances between all principal component (PC) scores from principal component analysis are shown in (a). Bray Curtis dissimilarity coefficients between pairs of templates are shown in (b). Error bars are standard errors of the means based on analyses of three replicate templates (*n* = 3).

Comparison of predicted and observed Euclidean distances between PC scores across templates produced a significant positive relationship (Figure 3a; Mantel test, using 10,000 randomisations *r* = 0.95, *P*<0.001). Comparisons of predicted and observed Bray Curtis dissimilarity coefficients across templates also produced a significant positive relationship (Figure 3b; Mantel test, using 10,000 randomisations; *r* = 0.96, *P*<0.001).

### Diversity measures

**Figure 4:**
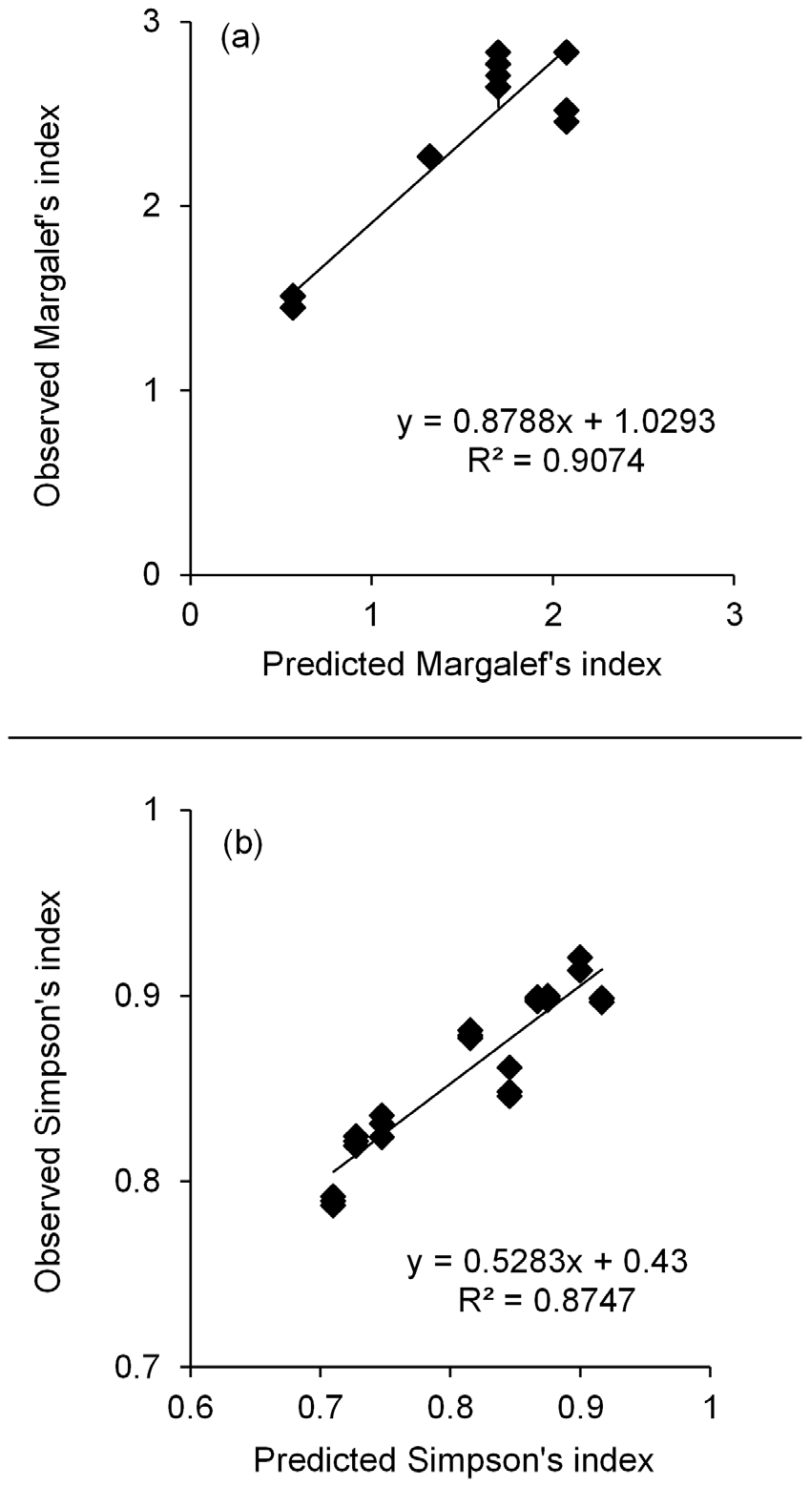
Relationship between predicted and observed a) Margalef's indices and b) Simpson's indices. Error bars are standard errors of the means based on technical replicates (replicate PCR and digestion) but are so small they are rarely visible (*n* = 3).

There was a significant linear relationship between predicted and observed Margalef's indices (*F*_1,25_ = 245, *P*<0.001, *R^2^* = 0.91; Figure 4a) showing the TRFLP protocol accurately estimated the differences in richness of the communities from quantitative data. Similarly, there was a significant positive relationship between the predicted and observed Simpson's indices (*F*_1,25_ = 175; *P*<0.001, *R^2^* = 0.87; Figure 4b) indicating quantitative use of the TRFLP data also accurately estimated differences in community evenness.

## Discussion

### Quantitative use of molecular data for compositional and diversity analyses

This study demonstrates that the TRFLP protocol tested can be used quantitatively to differentiate among communities of AM fungi with the same taxa but in different proportions (Figure 2). Comparing the predicted and observed TRFLP data using both PCA (Figure 3a) and Bray Curtis dissimilarity coefficients (Figure 3b) produced highly similar results, showing that relative abundance data generated by this technique can be used to accurately quantify relative differences between communities using commonly applied ordination methods. Moreover there was a strong positive relationship between the actual (predicted) and observed community diversities, indicating that both differences in taxon richness (Figure 4a) and evenness (Figure 4b) were accurately recovered. Bias in the different stages of the TRFLP protocol would disrupt these relationships. By empirically testing the limitations of this protocol we thereby demonstrate that quantitative use of TRFLP data is not affected by these biases.

In contrast to some other TRFLP protocols (e.g. ref. [29]), the results of the empirical analysis of experimental artificial community templates also indicate that the method used produces highly consistent results (as shown by the low variation between technical replicates; Figures 2 & 4). This indicates that stochastic processes affecting the amplification of individual genotypes are unlikely to be important and environmental samples need only be analysed once using this protocol. This highlights that TRFLPs can provide a robust, high throughput, powerful, repeatable method for studying differences in communities of AM fungi.

In addition to showing the strengths and limitations of the TRFLP protocol tested, the findings of this study have implications for other molecular analyses of these fungi. The TRFLP protocol included a PCR step using primers AM1 and NS31, which are also commonly used in other molecular analyses such as pyrosequencing [21], [36]. The results of this experiment imply that the amplification of these fungi using these primers was unbiased. This therefore supports the use of data on the relative abundances of AM fungal taxa generated by other methods using these primers. However, it should be noted that confirming this fully is beyond the scope of this study as there are many causes of PCR bias [22] and NGS may have different potential sources [37], [38]. The experimental approach described in this paper of generating artificial communities of known characteristics and testing protocols should therefore be adapted and repeated for different methods.

### Studies of individual taxa

Whilst this study suggests that using the abundance of TRFs can be of great use in studying AM fungi at the community level, it also shows extreme caution should be applied when using it to assess the presence and absence of individual taxa. TRFLP analysis over-estimated AM fungal richness in all samples (Figure 4a) due to the presence of TRFs that were not expected from the full digestion of the genotypes in the artificial templates (Table 1). These fragments corresponded to the sizes of predicted pseudo-TRFs, defined as non-terminal restriction fragments caused by incomplete digestion [32]. This observed under-digestion was unexpected and difficult to explain. The DNA concentrations of the purified PCR products restricted suggest tenfold more enzyme was used than should have been required for full digestion. Moreover, despite containing the same DNA quantity, the restriction of labeled PCR products of single sequence types exhibited very little under-digestion with over 99% of TRFs produced being the length predicted to be generated by complete digestion. This implies that the formation of pseudo-TRFs occurs more from mixed than single sequence template amplicons. It is possible that pseudo-TRFs were generated by three processes: 1) the presence of chimeric PCR products (i.e. sequences derived from more than one DNA template whereby a combination of two or more sequences are located on the same strand; [22]). This can disrupt restriction, as restriction loci may be swapped between different DNA sequences; 2) single stranded DNA formation as type II restriction enzymes only cleave double stranded DNA [32]; 3) Heteroduplex formation, where DNA molecules form when heterologous sequences bind to each other to form double stranded DNA, which can generate base pair mismatches in the enzyme recognition sequences, reducing efficiency of enzyme digestion [39], [40].

The observed pseudo-TRFs show that even when using TRFLPs qualitatively, with ‘diagnostic peaks' being used to indicate the presence of a particular genotype in a sample (e.g. as used in ref. [15]), care should be taken to ensure it is impossible to obtain false positives due to incomplete digestion. This could be achieved by *in silico* digestion of a sequence database from the study site, to ensure diagnostic TRFs are not used that are the same size as possible pseudo-TRFs. Moreover, the potential causes of pseudo-TRFs are not restricted to just TRFLP analysis, but happen as a result of errors in PCR. This highlights the importance of carefully checking richness estimates from any PCR-based method, and may account for the vast range in richnesses reported from NGS studies; different approaches to quality filtering of sequences (e.g. failure to remove all chimeras) may over inflate richness estimates [41]. This study also suggests that ideally, TRFLP analyses should also include empirical testing of the TRF sizes produced from individual fungal genotypes (as conducted in ref. [16]) as restriction of one genotype can unexpectedly produce more than one TRF and the estimated length of fragments can vary slightly from their predicted sizes based on their *in silico* digestions (Table 1), making matching TRFs to sequence databases difficult. This ‘TRF drift’ is most likely to be due to the effect of different fluorophores on the migratory properties of the fragments during capillary electrophoresis, causing the HEX and FAM labelled TRF fragments to move faster than the size standard, resulting in consistent underestimation of their sizes [42].

### Biological interpretation of molecular AM fungal community characterisation

Finally, when applying the conclusions of this experiment to future studies, standard methodological caveats on the biological interpretation of molecular analyses must also be applied. All TRFLP and NGS analyses measure the relative abundances of fungal taxa within samples, and should therefore not be used to infer differences in absolute abundances of taxa across samples. Furthermore, AM1 and NS31 are thought to amplify the vast majority of AM fungi, but exclude the Paraglomeraceae [43] so cannot be used to determine if there are differences in the communities of this group. Finally, nucleic acid based methods can only be used to assess changes in ‘genetic’ diversity and composition of AM fungal communities. ‘Inter-specific’ and temporal variation in gene copy number per unit growth in AM fungi [44], [45] mean it is impossible to use them to measure the abundance of fungal ‘species’ in terms of their biomass. However, as the concept of an AM fungal ‘species’ is still poorly defined [46], it is arguably preferable to measure these communities in terms of their genetic content anyway.

### Summary

This investigation has shown that TRFLPs can be used quantitatively to discriminate between different AM fungal communities containing the same genotypes in different relative abundances, and accurately measure the differences between them. In addition we have shown that the method is robust and reliable with little error in repeatability. The protocol described therefore has great potential to enhance our understanding of a wide variety of aspects of AM fungal ecology. The results also suggest there was no PCR bias in the protocol, supporting the use of AM fungal relative abundance data from other PCR based methods such as NGS.
